# Predicting Postoperative Complications After Cholecystectomy for Acute Cholecystitis: Comparative Performance of Disease-Specific and General Prognostic Scores

**DOI:** 10.3390/jcm15103595

**Published:** 2026-05-08

**Authors:** Marco Marcianò, Giuseppe Salamone, Giovanni Guercio, Bianca Vicari, Virginia Agostara, Isabella Campo, Claudia Militello, Giuseppina Orlando, Giuseppina Melfa, Gianfranco Cocorullo, Gregorio Scerrino

**Affiliations:** 1 General Surgery Unit, Department of Precision Medicine in Medical, Surgical and Critical Care (Me.Pre.C.C.), University of Palermo, 90133 Palermo, Italy; mm.94@hotmail.it (M.M.); giuseppe.salamone@unipa.it (G.S.); giuvanni.guercio@unipa.it (G.G.); virginia.agostara@you.unipa.it (V.A.); isabella.campo6@gmail.com (I.C.); claudia.militello@you.unipa.it (C.M.); giuseppina.orlando@policlinico.pa.it (G.O.); giuseppina.melfa@policlinico.pa.it (G.M.); gianfranco.cocorullo@unipa.it (G.C.); gregorio.scerrino@policlinico.pa.it (G.S.); 2 A.O.U.P. Paolo Giaccone, Via Liborio Giuffrè n.5, Edificio 4A, 90127 Palermo, Italy

**Keywords:** acute cholecystitis, cholecystectomy, risk-prediction, prognostic score, chole-risk, postoperative complications, length of stay

## Abstract

**Background:** Although early laparoscopic cholecystectomy represents the standard treatment for acute cholecystitis [AC], reliable preoperative risk stratification remains challenging. This study compared the discriminative performance of the following five prognostic scores: two disease-specific tools (Chole-Risk and a locally modified variant, Chole-Risk mod) and three general indices (POSSUM Physiological Score, APACHE II, and Charlson Comorbidity Index [CCI]) for predicting postoperative complications [POCs] and prolonged hospital stay. **Methods:** This single-center retrospective study included 211 consecutive patients who underwent cholecystectomy for AC between 2015 and 2024. Primary endpoint: the occurrence of any POC. Secondary endpoint: prolonged length of stay (LOS), defined as postoperative hospitalization exceeding the 75th percentile (>6 days). Discrimination was assessed using the area under the receiver operating characteristic curve (AUC), with pairwise comparisons performed using the DeLong test. Calibration was evaluated graphically, and clinical utility was explored through decision curve analysis. **Results:** POC occurred in 55 patients (26.1%), and prolonged LOS in 31 (14.7%). Chole-Risk mod showed the best discrimination (AUC 0.925) and the strongest association per one-standard-deviation increase (OR 16.60; 95% CI 9.49–43.56). Other scores showed lower performance as follows: POSSUM PS (AUC 0.732), CCI (0.712), Chole-Risk (0.699), and APACHE II (0.695). For prolonged LOS, Chole-Risk mod demonstrated the highest discrimination (AUC 0.869). Decision curve analysis confirmed a net clinical benefit for Chole-Risk mod across a broad range of decision thresholds. **Conclusions:** The modified Chole-Risk score showed the highest discrimination among the evaluated scores for predicting adverse outcomes after cholecystectomy for acute cholecystitis in this single-center exploratory cohort. These findings suggest that incorporating disease-specific variables may improve preoperative risk stratification, although prospective multicenter validation is warranted.

## 1. Introduction

Acute cholecystitis is among the most frequent surgical emergencies, responsible for a large share of unplanned admissions and emergency operating-room time [1,2,3].

In most cases (90–95%), acute cholecystitis results from cystic duct obstruction by gallstones, leading to gallbladder distension, ischemia, and secondary infection [4].

Early laparoscopic cholecystectomy, performed within 72 h of symptom onset or during the index admission, is now endorsed as the treatment of choice by the Tokyo Guidelines 2018 [5], the World Society of Emergency Surgery (WSES), and multiple meta-analyses, owing to shorter hospital stays, fewer recurrences, and comparable or lower complication rates relative to delayed surgery [6,7,8,9,10].

Nevertheless, the procedure carries measurable risks. Bile duct injury remains the most feared complication, occurring in 0.3–1.8% of cases and carrying mortality rates of up to 20% [11,12]. A higher incidence of bile duct injuries has been reported in the pediatric population, possibly reflecting lower surgical volume in children [13]. The principal mechanism is misidentification of ductal anatomy rather than a purely technical error, a risk amplified by inflammation, fibrosis, and the anatomical variability of the cystic duct, arterial supply, and gallbladder position [12,14,15]. The systematic adoption of the Critical View of Safety (CVS), introduced by Strasberg in 1995, significantly reduces the risk of bile duct injury without increasing operative time or conversion rates [16,17].

Recent large-scale analyses have further confirmed that male sex and acute cholecystitis itself are independent, non-modifiable risk factors for bile duct injury [18].

Beyond intraoperative biliary injuries, postoperative morbidity encompasses surgical-site infections, intra-abdominal collections, hemorrhage, and systemic complications [19,20]. Length of stay (LOS) has also been adopted as a surrogate marker of clinical complexity and a metric of organizational efficiency [21].

Several general prognostic scores, such as the American Society of Anesthesiologists Physical Status (ASA-PSs) classification, POSSUM, APACHE II, and the Charlson Comorbidity Index (CCI), have been widely used to stratify perioperative risk across surgical specialties [22,23,24,25]. However, these tools primarily reflect the patient’s systemic physiological reserve and comorbidity burden, while neglecting disease-specific variables related to biliary inflammation and surgical complexity. More recently, the Chole-Risk score was introduced by Di Martino et al. as a simple, preoperative tool designed expressly to predict complicated postoperative courses after early cholecystectomy for acute calculous cholecystitis [26]. The S.P.Ri.M.A.C.C. multicenter study subsequently provided prospective external validation, comparing Chole-Risk head-to-head with POSSUM PS, CCI, ASA-PS, APACHE II, and TG18 severity grading in 1253 patients across 79 centers [27].

Several questions remain open. First, it remains unclear whether the superiority of disease-specific scores over general indices is preserved in unselected, real-world cohorts that include all indications for cholecystectomy in the setting of acute cholecystitis. Second, most validation studies have focused on composite morbidity endpoints; the added ability of these tools to predict prolonged LOS, which is an outcome of considerable organizational relevance, has been less thoroughly investigated. Third, few studies have simultaneously assessed discrimination, calibration, and clinical utility (via decision curve analysis) to provide a comprehensive performance evaluation.

The aim of this study was to compare the predictive performance of the following five prognostic scores: Chole-Risk, a locally modified Chole-Risk variant (Chole-Risk mod), POSSUM Physiological Score, APACHE II, and CCI, in predicting postoperative complications and prolonged hospital stay after cholecystectomy for acute cholecystitis. Better risk prediction could inform surgical planning, patient counseling, and resource allocation in emergency biliary surgery.

We assessed discrimination, calibration, and clinical utility through decision curve analysis to determine whether disease-specific instruments offer better perioperative risk stratification than general severity indices.

## 2. Materials and Methods

### 2.1. Study Design and Ethical Considerations

This retrospective single-center observational cohort study was conducted at the AOUP “Paolo Giaccone”, University of Palermo, Palermo, Italy.

The study protocol was designed and reported in accordance with the Strengthening the Reporting of Observational Studies in Epidemiology (STROBEs) statement [28] and the Transparent Reporting of a multivariable prediction model for Individual Prognosis Or Diagnosis (TRIPOD) checklist [29].

In accordance with applicable local institutional procedures and national regulations, the study was not subject to formal ethical approval due to its retrospective, non-interventional design and the exclusive use of previously anonymized data collected during routine clinical care. The study was conducted in compliance with institutional guidelines for research involving anonymized datasets. No identifiable personal data were available to the investigators, and no additional procedures or changes in patient management were performed. Therefore, the requirement for individual informed consent was waived.

### 2.2. Population

**Figure 1:**
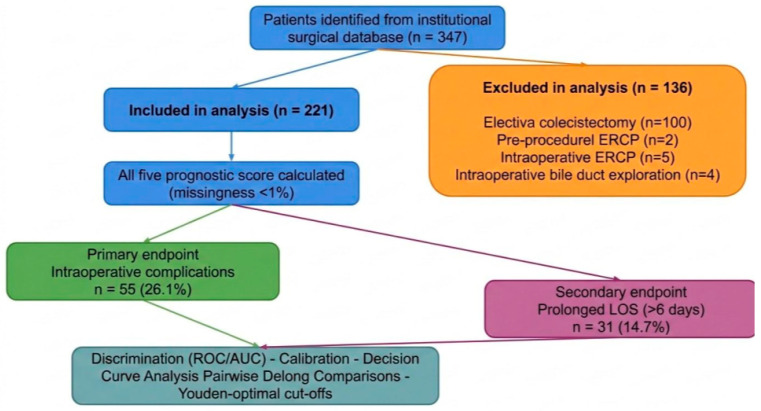
Study flowchart and analytic workflow. A total of 347 patients were identified from the institutional surgical database. After exclusions, 221 patients were included in the final analysis. Excluded cases included elective cholecystectomy, pre-procedural ERCP, intraoperative ERCP, and intraoperative bile duct exploration. For all included patients, five prognostic scores were calculated, with less than 1% missing data. The primary endpoint was intraoperative complications, observed in 55 patients, while the secondary endpoint was prolonged length of stay, defined as hospital stay longer than 8 days, observed in 31 patients. The prognostic performance of the scores was assessed through discrimination analysis using ROC/AUC, calibration analysis, decision curve analysis, pairwise DeLong comparisons, and identification of Youden-optimal cut-offs.

All consecutive patients who underwent cholecystectomy for acute cholecystitis between January 2015 and December 2024 were identified from a prospectively maintained institutional surgical database. A total of 347 patients were initially screened. The diagnosis of acute cholecystitis was established on the basis of clinical presentation, laboratory findings, and imaging studies consistent with the previously mentioned Tokyo Guidelines 2018. Inclusion was unrestricted with respect to the severity of cholecystitis, surgical approach, or patient comorbidity profile, thereby reflecting an unselected, real-world population. A total of 136 patients were excluded for the following reasons: elective cholecystectomy (*n* = 100), pre-procedural endoscopic retrograde cholangiopancreatography (ERCP) (*n* = 27), intraoperative ERCP (*n* = 5), and intraoperative common bile duct exploration (*n* = 4). Missingness among included patients was minimal (<1%) and did not appear systematic; a complete-case analysis was therefore deemed appropriate. The final analytical cohort comprised 211 patients (Figure 1).

### 2.3. Variables and Definitions

The following variables were extracted for each patient: age, sex, body mass index (BMI), comorbidities (coded as a binary variable), history of prior abdominal surgery, previous percutaneous cholecystostomy, signs of choledocholithiasis, intraoperative findings indicative of difficult cholecystectomy, Mirizzi syndrome, use of inotropes, and need for endoscopic retrograde cholangiopancreatography (ERCP). All dichotomous variables were coded as 1 = present and 0 = absent.

### 2.4. Prognostic Scores

The following five prognostic scores were calculated for each patient (Table 1):

Chole-Risk (pure): A preoperative score (range 0–4) developed by Di Martino et al. [26] to predict complicated postoperative courses after early cholecystectomy for acute calculous cholecystitis.

Chole-Risk modified (Chole-Risk mod): a post hoc exploratory modification constructed by adding the following three equally weighted binary variables to the original Chole-Risk score: acute-on-chronic cholecystitis, microlithiasis, and prior percutaneous cholecystostomy (1 point each; total range 0–7). This modification has not undergone independent validation and should be regarded as hypothesis-generating.

POSSUM Physiological Score (PS): The physiological component of the POSSUM system, integrating 12 preoperative physiological parameters [30].

APACHE II: A score originally designed for intensive-care prognostication, based on acute physiological derangements, age, and chronic health status [24].

Charlson Comorbidity Index (CCI): A weighted index quantifying the cumulative burden of chronic comorbidities [25].

### 2.5. Endpoints

The primary endpoint was the occurrence of any postoperative complication during the index hospitalization. Complications were graded according to the Clavien–Dindo classification and subsequently analyzed as a binary outcome (presence vs. absence of complications). Major complications were defined as Clavien–Dindo grade ≥ III.

The secondary endpoint was prolonged length of stay (LOS), defined as postoperative hospitalization exceeding the 75th percentile of the cohort distribution (>6 days).

### 2.6. Statistical Analysis

Continuous variables were described as mean ± standard deviation (SD) and median [interquartile range, IQR], and categorical variables as absolute frequencies and percentages. Between-group comparisons were performed using the Student t-test or Mann–Whitney U test for continuous variables and the chi-squared or Fisher exact test for categorical variables, as appropriate.

The association between each score and the binary endpoints was quantified by logistic regression, with results expressed as odds ratios (ORs) per one-standard-deviation (1-SD) increment to allow direct comparison across differently scaled instruments, together with 95% confidence intervals (CIs).

Discrimination was evaluated by receiver operating characteristic (ROC) curve analysis with calculation of the area under the curve (AUC), and 95% confidence intervals were estimated using 2000 bootstrap resamples. Pairwise AUC comparisons were performed using the nonparametric DeLong test [31]. In view of the moderate class imbalance of both endpoints, precision–recall (PR) curves and average precision (AP) were also computed.

For each score, clinically actionable cut-offs were identified using the Youden index, and the corresponding sensitivity, specificity, positive predictive value (PPV), negative predictive value (NPV), and accuracy were reported.

Model calibration was assessed graphically via calibration plots on ten decile bins of predicted risk. Clinical utility was evaluated by decision curve analysis (DCA), estimating the net benefit across a range of clinically relevant threshold probabilities [32].

Analyses were performed using Python 3.12 (scikit-learn 1.4, scipy 1.11). A two-sided *p* value < 0.05 was considered statistically significant.

### 2.7. AI Statement

During manuscript preparation, the authors used Claude Opus 4.6 to assist with English language refinement and the preparation of graphical outputs. The AI tool was not used as a substitute for authorship, scientific judgment, or interpretation of the data. All generated material was carefully checked, edited, and approved by the authors, who assume full responsibility for the final content.

## 3. Results

A total of 211 patients met the inclusion criteria and constituted the analytical cohort. Their baseline characteristics are summarized in Table 2. The mean age was 53.4 ± 17.8 years, with a near-equal sex distribution (51.2% female). The median BMI was 26.0 kg/m^2^ [IQR 23.6–29.4]. Acute-on-chronic cholecystitis was present in 20.9% and microlithiasis in 24.6% of patients. Relevant surgical history included prior abdominal surgery in 37.9% and previous percutaneous cholecystostomy in 4.3%. Comorbidities were documented in 23.2%. Signs of choledocholithiasis were present in 75.8%, intraoperative findings suggestive of difficult cholecystectomy in 29.4%, and ERCP was performed in 18.0% of cases.

The median postoperative stay was 3.0 days [IQR 2.0–4.0]. Postoperative complications occurred in 55 patients (26.1%), while 31 patients (14.7%) experienced prolonged LOS exceeding the 75th percentile threshold (6 days).

### 3.1. Association Between Scores and Outcomes

All five scores demonstrated a statistically significant association with postoperative complications (all *p* < 0.001; Table 3). For prolonged LOS, the Chole-Risk mod again showed the strongest association (*p* < 0.001), while the remaining scores also reached statistical significance (*p* = 0.004 to *p* = 0.014). For postoperative complications, the Chole-Risk mod exhibited the strongest association per 1-SD increment (OR 16.60; 95% CI 9.49–43.56), followed by POSSUM PS (OR 2.60; 1.89–3.71), CCI (OR 2.31; 1.73–3.25), Chole-Risk (OR 2.30; 1.61–3.49), and APACHE II (OR 1.92; 1.43–2.80). For prolonged LOS, the Chole-Risk mod again demonstrated the strongest association (OR 3.84; 2.67–7.05), followed by CCI (OR 1.64; 1.16–2.41), Chole-Risk (OR 1.58; 1.12–2.40), APACHE II (OR 1.53; 1.11–2.18), and POSSUM PS (OR 1.52; 1.09–2.19).

### 3.2. Discrimination

**Figure 2:**
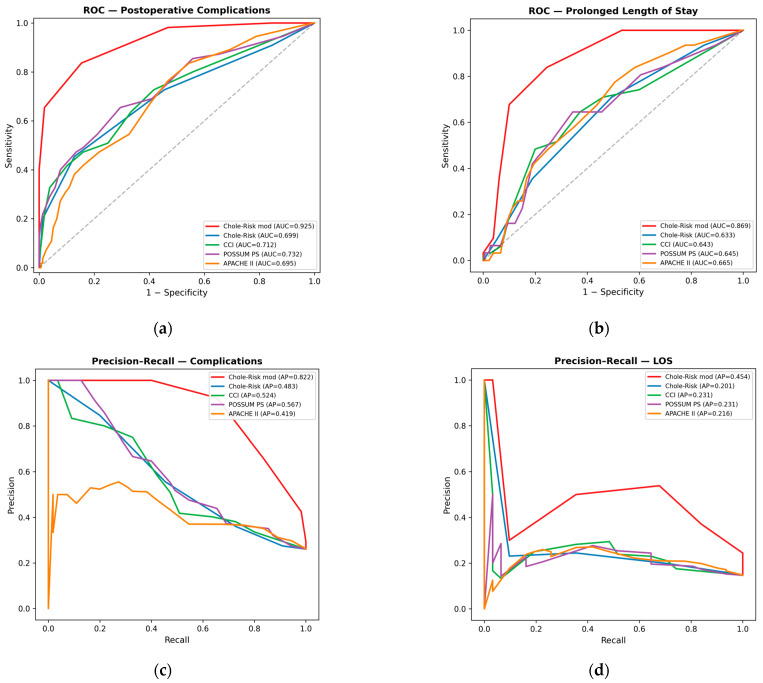
Discriminative performance of the evaluated prognostic scores for postoperative complications and prolonged length of stay: ROC and precision–recall analyses for each of the five evaluated scores. Sub-panels: (**a**) ROC curves for postoperative complications; (**b**) ROC curves for prolonged LOS (>6 postoperative days); (**c**) precision–recall curves for complications; (**d**) precision–recall curves for prolonged LOS. Each curve represents one score; AUC (for ROC) and AP (for PR) values are shown in the legend. The diagonal dashed line in ROC panels represents random classification. CCI, Charlson Comorbidity Index; LOS, length of stay; PS, physiological score; AUC, area under the curve; AP, average precision.

The prediction of complications was evaluated by ROC analysis. The Chole-Risk mod achieved the highest AUC of 0.925 (95% CI 0.884–0.960), exceeding Chole-Risk (0.699; 0.612–0.781), CCI (0.712; 0.628–0.788), POSSUM PS (0.732; 0.651–0.810), and APACHE II (0.695; 0.616–0.772) (Figure 2a). Pairwise DeLong tests confirmed that the Chole-Risk mod showed significantly higher AUC than all other scores (all *p* < 0.001). No significant differences were found among the remaining four scores.

For the prediction of prolonged LOS, the Chole-Risk mod again demonstrated the highest discrimination (AUC 0.869; 0.815–0.923), exceeding APACHE II (0.665; 0.560–0.760), POSSUM PS (0.645; 0.532–0.747), CCI (0.643; 0.533–0.750), and Chole-Risk (0.633; 0.535–0.743) (Figure 2b). DeLong comparisons confirmed that the Chole-Risk mod showed significantly higher AUC than all other scores for this endpoint as well (all *p* < 0.001). No significant pairwise differences were detected among the remaining four scores.

Considering the moderate class imbalance (complications: 26.1%; prolonged LOS: 14.7%), precision–recall curves were also generated. For complications, the Chole-Risk mod achieved the highest average precision (AP 0.822), followed by POSSUM PS (0.567), CCI (0.524), Chole-Risk (0.483), and APACHE II (0.419). For prolonged LOS, the Chole-Risk mod again led (AP 0.454), followed by CCI (0.231), POSSUM PS (0.231), APACHE II (0.216), and Chole-Risk (0.201) (Figure 2c,d).

### 3.3. Cut-Off Identification

Youden-index-derived cut-offs and their corresponding diagnostic performance metrics are presented in Table 4. For complications, the Chole-Risk mod ≥3 yielded a sensitivity of 83.6%, a specificity of 84.6%, and an NPV of 93.6%, offering strong discrimination as both a rule-out and rule-in threshold. For prolonged LOS, the Chole-Risk mod ≥3 showed a sensitivity of 83.9% and an NPV of 96.5%, supporting its use in perioperative triage.

### 3.4. Calibration

**Figure 3:**
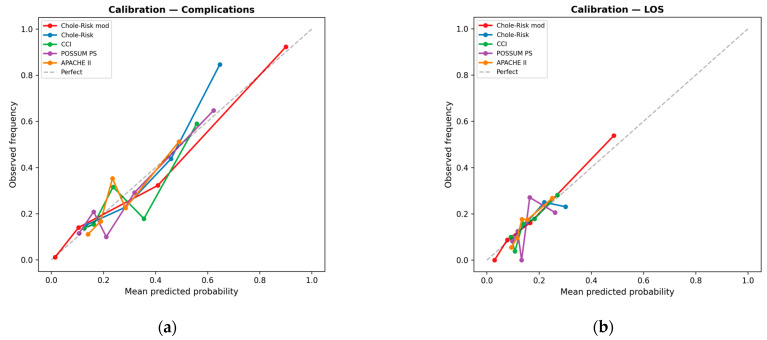
Calibration plots: (**a**) Postoperative complications. (**b**) Prolonged LOS. For each score, logistic regression-derived predicted probabilities were grouped into quantile bins; observed event frequencies are plotted against mean predicted probabilities. The dashed diagonal represents perfect calibration. CCI, Charlson Comorbidity Index; LOS, length of stay; PS, physiological score.

Calibration plots demonstrated good agreement between predicted probabilities and observed event rates for the Chole-Risk instruments, particularly for the complications endpoint, with data points clustering near the ideal diagonal. General scores exhibited greater dispersion from the calibration line, suggesting suboptimal calibration in this specific surgical context (Figure 3a,b).

### 3.5. Decision Curve Analysis

**Figure 4:**
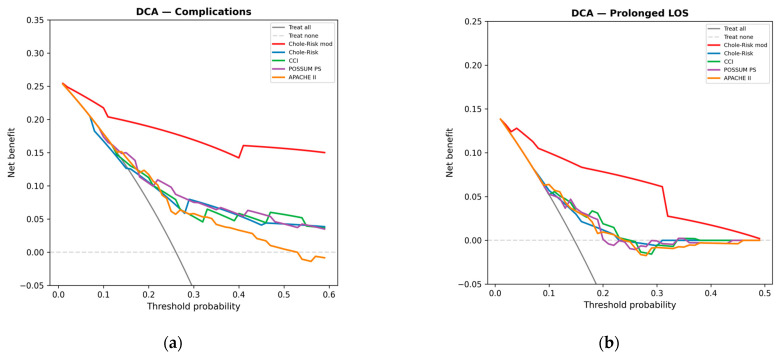
Decision curve analysis: (**a**) Postoperative complications. (**b**) Prolonged LOS. Net benefit is plotted against threshold probability for each score and for the treat-all and treat-none reference strategies. A score is clinically useful at a given threshold when its curve lies above both reference lines. CCI, Charlson Comorbidity Index; LOS, length of stay; PS, physiological score.

The DCA demonstrated that, for the prediction of postoperative complications, the Chole-Risk mod provided the highest net benefit across a broad range of clinically plausible threshold probabilities (approximately 10–50%), consistently exceeding the treat-all and treat-none reference strategies as well as all other scores. For prolonged LOS, the Chole-Risk mod similarly showed the highest net benefit across the examined threshold range (Figure 4a,b).

## 4. Discussion

The present study provides a comparative evaluation of five prognostic scores for predicting postoperative complications and prolonged hospital stay after cholecystectomy for acute cholecystitis. The main finding is that the modified Chole-Risk score showed the highest discrimination in this single-center cohort compared with both the original Chole-Risk and widely used general prognostic indices such as POSSUM PS, APACHE II, and the Charlson Comorbidity Index. The original Chole-Risk score, however, performed comparably to the general indices. These results suggest that the incorporation of additional disease-specific variables reflecting biliary pathology complexity, specifically acute-on-chronic cholecystitis, microlithiasis, and prior percutaneous cholecystostomy, may improve preoperative risk stratification.

Clinically, these data suggest that postoperative morbidity after cholecystectomy for acute cholecystitis depends more on local biliary pathology than on the patient’s overall physiological status. General-purpose scores capture comorbidity burden and physiological reserve, both relevant to perioperative risk but not specific to biliary disease. Other disease-oriented models targeting operative difficulty have been proposed, including the preoperative risk score by Nassar et al. [32].

However, they do not incorporate variables specific to gallbladder disease such as inflammatory severity, anatomical distortion, or indicators of technically demanding dissection, all of which directly influence operative difficulty. Anatomical variability of the cystic duct and biliary tree further increases misidentification risk during dissection [33].

The Chole-Risk score was designed to incorporate these disease-related features, and the results of the present analysis support its rationale.

The magnitude of this difference is clinically meaningful. In our cohort, the Chole-Risk mod achieved excellent discriminative performance for postoperative complications (AUC 0.925), exceeding that of the best general index (POSSUM PS, AUC 0.732) by approximately 19 percentage points and demonstrating a stronger association with the outcome when standardized per one standard deviation increment (OR 16.60 vs. 2.60). Pairwise DeLong comparisons confirmed the statistical significance of these differences (all *p* < 0.001). These observations align with the rationale underlying the development of the original Chole-Risk score [26] and are concordant with the findings of the S.P.Ri.M.A.C.C. prospective multicenter validation study, which also demonstrated the superiority of disease-specific risk stratification compared with general indices [27].

The present analysis extends prior evidence in two ways. First, we studied an unselected population undergoing cholecystectomy for acute cholecystitis in a real-world emergency setting, which strengthens external applicability. Second, we went beyond ROC analysis to evaluate calibration and clinical utility through decision curve analysis.

On decision curve analysis, Chole-Risk-based instruments provided a higher net benefit across a wide range of threshold probabilities, suggesting potential for practical decision support in perioperative management [34].

For prolonged length of stay, all scores showed lower and more homogeneous discrimination. This likely reflects the multifactorial nature of hospital stay, which depends not only on clinical severity but also on bed availability, discharge policies, and rehabilitation logistics [21]. No purely clinical score can fully capture this complexity.

Beyond global measures of predictive accuracy, the identification of operational cut-offs represents an important step toward practical implementation. In our dataset, a Chole-Risk mod score below three identified patients with a low probability of both postoperative complications (NPV 93.6%) and prolonged hospital stay (NPV 96.5%), supporting its potential use as a rule-out threshold in perioperative triage. In a clinical workflow, such patients could be considered candidates for fast-track or short-stay protocols. Conversely, a Chole-Risk mod score ≥3 was associated with high sensitivity for both endpoints (83.6% and 83.9%, respectively), suggesting a role in identifying patients who may benefit from enhanced perioperative monitoring, early multidisciplinary involvement, proactive resource allocation, and preoperative counseling regarding the likelihood of postoperative complications or an extended hospital stay. Such a stratified approach could contribute to improved bed management and more efficient use of hospital resources, particularly in high-volume emergency surgery settings. However, these thresholds were derived from a single-center cohort and require prospective validation before clinical adoption.

Strengths of the study include the use of a consecutive real-world cohort, comparison of five scores within a single analytical framework, and the assessment of discrimination, calibration, and clinical utility in parallel.

Evaluating both postoperative complications and prolonged hospital stay adds practical value, as these endpoints are of direct interest to clinicians and hospital administrators.

Several limitations should nevertheless be acknowledged. The retrospective single-center design introduces potential selection and information biases and may limit the generalizability of the findings to other healthcare systems and geographic regions. Local practice patterns, including perioperative protocols, indications for surgery, and conversion thresholds, may differ substantially across institutions. The long inclusion period (2015–2024) may have introduced temporal heterogeneity in clinical management, as evolving adoption of guidelines such as the Tokyo Guidelines 2018 and changes in antibiotic protocols or use of percutaneous cholecystostomy could have influenced both outcomes and model performance over time. The sample size, while adequate for the primary analysis, was moderate (*n* = 211). In addition, postoperative complications were analyzed as a binary outcome without formal severity grading according to a standardized classification system, which precluded the evaluation of score performance for major versus minor complications. The length-of-stay endpoint is particularly susceptible to non-clinical determinants, including institutional discharge policies, bed availability, social support structures, and rehabilitation logistics, which may vary substantially across centers and over time and are not captured by any purely clinical score. Finally, the modified Chole-Risk score represents a post hoc, exploratory adaptation that has not undergone independent external validation and should therefore be considered hypothesis-generating rather than a clinically ready prediction tool. Prospective multicenter validation is required before it can be recommended for widespread clinical adoption.

External validation in prospective, multicenter cohorts across different healthcare settings is the logical next step.

Disease-specific risk stratification, particularly through the modified Chole-Risk score, may be better suited to predicting adverse outcomes after cholecystectomy for acute cholecystitis than general indices. Prospective multicenter studies are needed to validate these findings and to clarify how such tools can be integrated into routine surgical workflows.

## 5. Conclusions

In a real-world cohort of patients undergoing cholecystectomy for acute cholecystitis, the modified Chole-Risk score showed the highest discrimination for predicting postoperative complications and prolonged hospital stay in this single-center exploratory cohort. These findings suggest that incorporating variables reflecting biliary disease complexity may improve preoperative risk stratification, although external validation is needed.

Incorporating disease-specific prognostic tools into clinical workflows could improve risk stratification and resource planning in emergency biliary surgery. Prospective multicenter validation is required to confirm these results and to facilitate broader clinical implementation.

## Figures and Tables

**Table 1:** Summary of evaluated prognostic score components.

Score	Components	Range	Reference
Chole-Risk	Male sex (1 pt), comorbidities (1 pt), signs of choledocholithiasis (1 pt), signs of difficult cholecystectomy (1 pt)	0–4	[26]
Chole-Risk mod	Chole-Risk (0–4) + acute-on-chronic cholecystitis (1 pt) + microlithiasis (1 pt) + prior percutaneous cholecystostomy (1 pt)	0–7	Present study
POSSUM PS	Twelve physiological parameters (age, cardiac signs, respiratory signs, blood pressure, pulse, GCS, hemoglobin, white cell count, urea, sodium, potassium, ECG)	12–88	[30]
APACHE II	Twelve acute physiological variables + age points + chronic health evaluation	0–71	[24]
CCI	Nineteen weighted comorbidity categories (MI, CHF, PVD, CVA, dementia, COPD, connective tissue disease, peptic ulcer, liver disease, diabetes, hemiplegia, CKD, solid tumor, leukemia, lymphoma, AIDS)	0–37	[25]

**Table 2:** Baseline characteristics of the study cohort (*n* = 211).

Variable	Total (*n* = 211)	Median [IQR]/Note
Sex = Female, *n* (%)	108 (51.2%)	—
Sex = Male, *n* (%)	103 (48.8%)	—
Age (years), mean ± SD	53.38 ± 17.83	56.0 [40.0–68.0]
BMI (kg/m^2^), mean ± SD	27.12 ± 5.28	25.97 [23.6–29.4]
POSSUM PS, mean ± SD	16.48 ± 3.94	15.0 [13.0–18.0]
APACHE II, mean ± SD	8.01 ± 5.25	7.0 [4.0–10.0]
CCI, mean ± SD	2.50 ± 2.76	1.0 [0.0–4.0]
Chole-Risk (0–4), mean ± SD	1.66 ± 1.08	1.0 [1.0–2.0]
Chole-Risk mod, mean ± SD	2.16 ± 1.58	2.0 [1.0–3.0]
Postoperative days (PODs), mean ± SD	4.69 ± 7.21	3.0 [2.0–4.0]
Acute-on-chronic cholecystitis, *n* (%)	44 (20.9%)	—
Microlithiasis, *n* (%)	52 (24.6%)	—
Prior abdominal surgery, *n* (%)	80 (37.9%)	—
Prior percutaneous cholecystostomy, *n* (%)	9 (4.3%)	—
Comorbidities, *n* (%)	49 (23.2%)	—
Signs of choledocholithiasis, *n* (%)	160 (75.8%)	—
Signs of difficult cholecystectomy, *n* (%)	62 (29.4%)	—
Mirizzi syndrome, *n* (%)	5 (2.4%)	—
Inotrope use, *n* (%)	11 (5.2%)	—
ERCP performed, *n* (%)	38 (18.0%)	—

BMI, body mass index; CCI, Charlson Comorbidity Index; ERCP, endoscopic retrograde cholangiopancreatography; PODs, postoperative days; IQR, interquartile range; PS, physiological score; SD, standard deviation.

**Table 3:** Association (OR per 1-SD) and discrimination (AUC) of each score for the two endpoints. All ORs *p* < 0.001 for postoperative complications. For prolonged LOS, all associations *p* < 0.05 (Chole-Risk mod *p* < 0.001; others *p* = 0.004–0.014).

Score	Outcome	OR (1-SD)	95% CI	AUC	AUC 95% CI
Chole-Risk mod	Complic.	16.60	9.49–43.56	0.925	0.884–0.960
Chole-Risk	Complic.	2.30	1.61–3.49	0.699	0.612–0.781
CCI	Complic.	2.31	1.73–3.25	0.712	0.628–0.788
POSSUM PS	Complic.	2.60	1.89–3.71	0.732	0.651–0.810
APACHE II	Complic.	1.92	1.43–2.80	0.695	0.616–0.772
Chole-Risk mod	LOS > p75	3.84	2.67–7.05	0.869	0.815–0.923
Chole-Risk	LOS > p75	1.58	1.12–2.40	0.633	0.535–0.743
CCI	LOS > p75	1.64	1.16–2.41	0.643	0.533–0.750
POSSUM PS	LOS > p75	1.52	1.09–2.19	0.645	0.532–0.747
APACHE II	LOS > p75	1.53	1.11–2.18	0.665	0.560–0.760

AUC, area under the receiver operating characteristic curve; CCI, Charlson Comorbidity Index; CI, confidence interval; LOS, length of stay; OR, odds ratio; PS, physiological score; SD, standard deviation.

**Table 4:** Diagnostic performance at the Youden-optimal cut-off for each score and endpoint.

Outcome	Score	Cut-Off	Sensitivity (%)	Specificity (%)	PPV (%)	NPV (%)	Accuracy (%)	AP
Complic.	Chole-Risk	≥3	45.5	87.2	55.6	81.9	76.3	0.483
Complic.	Chole-mod	≥3	83.6	84.6	65.7	93.6	84.4	0.822
Complic.	POSSUM PS	≥17	65.5	70.5	43.9	85.3	69.2	0.567
Complic.	APACHE II	≥7	76.4	53.2	36.5	86.5	61.6	0.419
Complic.	CCI	≥6	41.8	89.7	59.0	81.4	75.8	0.524
LOS > p75	Chole-Risk	≥2	71.0	50.6	19.8	91.0	53.6	0.201
LOS > p75	Chole-mod	≥3	83.9	75.6	37.1	96.5	76.8	0.441
LOS > p75	POSSUM PS	≥17	64.5	65.6	24.4	85.3	65.4	0.231
LOS > p75	APACHE II	≥7	77.4	49.4	20.9	92.7	53.6	0.216
LOS > p75	CCI	≥5	49.0	83.8	49.0	83.8	75.4	0.231

AP, average precision; CCI, Charlson Comorbidity Index; LOS, length of stay; NPV, negative predictive value; PPV, positive predictive value; PS, physiological score.

## Data Availability

The data supporting the reported results are not publicly available due to privacy and institutional restrictions, as they derive from retrospective clinical data collected during routine care. Fully anonymized data may be made available by the authors upon reasonable request, subject to institutional approval and applicable data protection regulations.
